# Advances in the synthesis of Fe-based bimetallic electrocatalysts for CO_2_ reduction

**DOI:** 10.1039/d4ra08833f

**Published:** 2025-03-18

**Authors:** Ayesha Zafar, Adnan Majeed, Abdul Ahad, Muhammad Adnan Iqbal, Tanveer Hussain Bokhari, Zanira Mushtaq, Shahzaib Ali

**Affiliations:** a Department of Chemistry, University of Agriculture Faisalabad Faisalabad-38000 Pakistan adnan.iqbal@uaf.edu.pk aayeshazafar99@gmail.com 2021ag2578@uaf.edu.pk zanira14802@gmail.com zaibsmd@gmail.com; b Organometallic and Coordination Chemistry Laboratory, University of Agriculture Faisalabad Faisalabad-38000 Pakistan; c Department of Chemistry, Government College University Faisalabad Faisalabad-38000 Pakistan aa.ahad9998@gmail.com tanveer.bokhari@yahoo.com

## Abstract

Achieving carbon neutrality and slowing down global warming requires research into  the electrochemical CO_2_ reduction reaction (CO_2_RR), which produces useful compounds. Utilizing renewable energy to meet carbon-neutral energy goals produces single-carbon (C_1_) and multi-carbon (C_2+_) goods. Efficient and selective electrocatalysts are essential to advancing this revolutionary technology; bimetallic Fe-based catalysts work better than their monometallic counterparts because multiple metals work synergistically to reduce CO_2_ levels. A thorough summary of recent developments in the synthesis of Fe–X bimetallic catalysts will be provided in this review, with an emphasis on key performance indicators like stability, faradaic efficiency, potential, current density, and primary product production. In addition, this analysis will look at representative instances of Fe bimetallic catalysts that are well-known for their selectivity in generating particular alcohols and hydrocarbons, clarifying the mechanics behind CO_2_ reduction, pointing out existing difficulties, and examining the potential of electrosynthesis processes in the future.

## Abstract 2

Achieving carbon neutrality and mitigating global warming through efficient CO_2_ reduction, by utilizing synergistic bimetallic Fe-based catalysts for enhanced electrochemical performance and selectivity.

## Graphical abstract

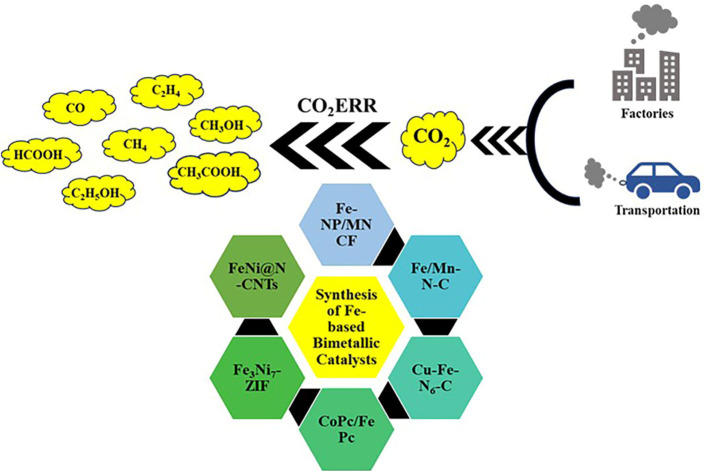

## 1. Introduction

Over the past 170 years, human activity has caused a sharp rise in CO_2_ emissions, which has resulted in ocean acidification and global climate change.^1,2^ Terrestrial ecosystems only absorb around 30% of CO_2_ produced by humans annually, which is insufficient to offset anthropogenic emissions, and the amount of CO_2_ in the atmosphere has dramatically increased in recent decades, reaching 400 ppm for the first time in human history. Since the late 1950s, the Mauna Loa Observatory in Hawaii has been continually monitoring atmospheric CO_2_, and as of 2024, CO_2_ concentrations are approximately 417 ppm. One of the main causes of climate change, this represents a sharp rise from pre-industrial levels of about 280 ppm.^3–5^ According to predictions, if present emission trends continue, CO_2_ levels might surpass 450 ppm by 2030. If immediate mitigation measures are not taken, some models forecast significantly higher concentrations.^6^ Over 450 ppm could dramatically raise the probability of catastrophic climate consequences, including more severe and frequent heatwaves, rising sea levels, and disturbances to ecosystems and food security.^7^

Although CO_2_ is a very stable molecule that is usually inert, it can undergo electrochemical activation and be transformed into reduced products through the CO_2_ reduction reaction (CO_2_RR) with the help of protons in solution and appropriate cathodic reduction potentials.^8,9^ Several methods have been suggested to change CO_2_ into value-added products, such as chemical transformation,^10,11^ reduction by photocatalysis,^12–14^ reduction by electrocatalysis,^15,16^ thermal catalysis,^17,18^ photothermal catalysis,^19^ as well as biological conversion.^20,21^ The simplicity, mild reaction conditions, environmental compatibility, and possible integration with energy from renewable sources of electrocatalytic CO_2_ reduction (eCO_2_RR) make it stand out among these applications. Since CO_2_ has a linear, symmetrical structure with a zero-dipole moment, it is stable and challenging to activate in electrocatalytic reduction. Because of the molecule's symmetry, the opposing dipoles of the C=O bonds cancel each other out, resulting in a low electron density around the molecule's center, especially close to the carbon atom. The molecule's general lack of a dipole moment is a result of the decreased electron density surrounding the carbon center. It is more difficult to activate CO_2_ due to its low electron density. Effective electrocatalytic reduction requires the catalyst to capture and hold CO_2_ molecules long enough for the reaction. However, CO_2_ lacks a strong dipole and other reactive properties, making it more difficult to activate for the reduction process. This demands extremely effective catalysts that can change CO_2_'s electron density or aid in breaking its strong C=O bonds.^22,23^ A significant overpotential is needed for CO_2_ activation due to the dissociation energy needed to break the C=O bond, which is more than 750 kJ mol^−1^, leading to poor energy efficiency and high operational costs.^24^ The shale gas revolution of the last twenty years, especially the large amounts of ethane (3–12% fraction) from shale gas, has changed the dynamics of the global energy market and produced an excess of ethane despite its relatively low market price, which is especially noticeable in the United States.^25,26^ Studies on CO_2_ reduction for CO and carbon-based energy sources^27–29^ have gained increasing attention owing to rising atmospheric CO_2_ levels and expanding energy demands, with a focus on finding inexpensive, efficient, and selective catalysts.^30–32^ These catalysts include homo-bimetallic sites (Fe–Fe, Co–Co, Ni–Ni, Cu–Cu) that indicate enhanced reactivity in comparison to monometallic counterparts, while hetero-bimetallic catalysts remain relatively overlooked.^33–41^

Efficient CO_2_ reduction catalyst design is hard due to stability, huge potentials,^29,42^ restricted solubility, competing HER, and slow kinetics.^43–46^ Reduced activation barriers are critical for improving electrocatalyst efficiency and selectivity. Despite the beneficial features of iron-group metallic alloys and compounds, their efficacy as catalysts in CO_2_ reduction remains insufficient due to low activity and stability.^47,48^ Because Fe is so readily available, it is essential to build highly efficient Fe–N–C catalysts. With the abundance of iron, there is a need to produce these catalysts. Fe-porphyrins treated with phenolic groups showed remarkable CO faradaic yields exceeding 90% without degradation, emphasizing Fe–N_4_ sites in macrocycles as active centers.^49^ The incorporation of Fe atomically into nitrogen-doped carbon substrates, such as Fe–N–C catalysts, has demonstrated remarkable catalytic reactivity towards CO_2_ reduction to CO, with Fe–N_4_ sites largely studied as active sites in several investigations.^50–53^ Fe species provide dynamic surface manipulation, which is critical for understanding structure dynamics and rational catalyst designing in CO_2_ electro-reduction reaction (CO_2_ERR).^54–56^ Recent studies reveal that heteroatom inclusion in carbon support alters the electrical environment, allowing tailored Fe sites to lower the energy of activation limitations in electrocatalysis.^57^ Fe or Cu-based metals/alloys are widely used as catalysts in CO_2_ reduction; Fe has significant catalytic activity and a minimal energy barrier, whereas Cu has excellent CO_2_ adsorption traits and resistance to coking.^58–61^ In addition to heteroatom inclusion, the use of a second metal atom in Fe-based materials also termed dual-atomic catalysts (DACs) improves catalytic activity synergistically. CO_2_ reduction relies on DACs, while Ru, Fe, Mn-based homogeneous, and Cu-based heterogeneous catalysis provide viable alternatives.^62–72^ The adsorbate–metal surface interaction in Fe–N–C single-atom catalysts (also known as SACs) is influenced by the shift of the d-band center.^73^ Determining the intensity and kind of these interactions between molecules is largely dependent on this change. Consequently, it has a major effect on the catalytic activity. Integrating heteronuclear metal atoms such as Ni, Co, and Zn permits electronic structure adjustment, which facilitates adsorbate absorption as well as desorption on the surface of the catalyst.^74–76^ Recently, multiple reviews have investigated the CO_2_ERR, spanning diverse catalysts like as copper–palladium nanoalloys,^9^ Cu-based nanocrystals,^77^ bimetallic chalcogenides,^78^ bimetallic catalysts with atomic sites,^79^ Bi-based,^80^ Ni-based,^81^ Sn-based,^82^ and carbide-based bimetallic catalysts.^83^ Fe-based bimetallic electrocatalysts are superior to other metals because they are more affordable, widely available, and have better selectivity for CO_2_ reduction products. When it comes to stability, efficiency, and scalability, they can perform better than single-metal catalysts like Cu. There is currently no review that provided in-depth analysis of the research on Fe-based bimetallic electrocatalysts for CO_2_ reduction. Given the growing importance of electrolytic CO_2_ reduction, the performance of Fe-based bimetallic catalysts merits a thorough examination. This research focuses on the production, implementation, and mechanistic understanding of these catalysts in CO_2_ electrocatalysis, covering a wide spectrum of product forms. Furthermore, the study highlights the problems and opportunities in developing and comprehending Fe-based bimetallic electrocatalysts, which offer useful insights for future research paths in this sector.

## 2. CO_2_ reduction pathways over Fe-based bimetallic electrocatalysts

**Fig. 1:**
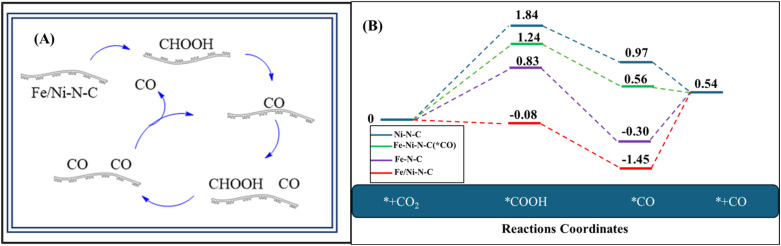
(A) Proposed paths for reduction of CO_2_ into CO over Fe/Ni–N–C. (B) Using DFT, the electrochemical reduction of CO_2_ to CO on Fe–N–C, Ni–N–C, and Fe/Ni–N–C with and without adsorbed *CO was represented by a free energy diagram.

Fe/Ni–N–C materials were used as electrocatalytic reduction (ECR) catalysts by Huiying Tian and coworkers. These substances were utilized to speed up the electrochemical processes that produce CO.^84^Fig. 1A illustrates the CO_2_ reduction into CO over the Fe/Ni–N–C catalyst. For the catalytic reduction of CO_2_ into CO, the Fe/Ni–N–C catalyst provides sites for CO_2_ to bind, and Fe/Ni acts as active centers for the reduction. For binding, CO_2_ accepts electrons and protons from the electrolyte solution and converts them into intermediate CO precursors such as CHOOH. This reaction is typically completed in two steps CO_2_ → COOH → CO* as mentioned in Fig. 1. The Fe/Ni–N–C electrocatalyst achieved an impressive (faradaic efficiency of CO) FE_CO_ of 92.9% at −0.677 V *vs.* (reversible hydrogen electrode) RHE, indicating great efficiency. The system retained an elevated current density and faradaic efficiency for the generation of CO (FE_CO_) when applied in a continuous flow cell at scale, holding onto over 89% shortly after 40 hours of electrolysis. Because of the binary metals combined effect, charge transfer rates were increased, resulting in favorable kinetics and long-term, effective electrochemical performance.^57^

To thoroughly investigate the combined effect of Ni–N–C, Fe–N–C, and Fe/Ni–N–C on ECR, DFT studies were performed utilizing the computational hydrogen electrode technique.^85^ In electrocatalytic CO_2_ reduction, the Fe atom's electrical properties and catalytic action are largely determined by its spin orientation.^86,87^ Fe can exist in both high and low spin states in bimetallic Fe-based catalysts, which affects the electron distribution in the d-orbitals and changes the CO_2_ and intermediate adsorption strength.^88^ While a low-spin level may produce more stable, less reactive configurations, a high-spin state can increase the activation of CO_2_ by offering more accessible electron states.^89^ The impact of these spin arrangements on the CO_2_ reduction reaction mechanism and efficiency is clarified using DFT simulations. The major catalytic sites, Me–N_4_ motifs, were used as single-site models based on prior investigations.^51,90^ The electrocatalytic reduction (ECR) process involved typical two-electron and two-proton transfer reactions, culminating in the creation of *COOH and *CO intermediates. The symbol asterisk (*) represents the active site. Ni–N–C, Fe–N–C, and Fe/Ni–N–C optimized geometries served as computational models for the investigation.^91,92^ On Fe–N–C sites, the rate-determining step was *CO → CO_(g)_, while on Ni–N–C sites, it was CO_2(g)_ → *COOH. Fe/Ni–N–C generated *COOH intermediates easily, while *CO desorption was difficult. Fe/Ni–N–C adsorbed with *CO intermediates had a much smaller free energy shift for the rate-determining phase *CO → CO_(g)_, indicating easier desorption. This shows that Fe/Ni–N–C provides more active sites by efficiently combining the benefits of Ni–N–C and Fe–N–C sites, increasing CO generation catalytic activity. CO_2_ adsorption on Fe–Ni bimetallic sites, electron and proton transfer pathways to form *COOH within *CO intermediate, and subsequent CO_(g)_ desorption to regenerate Fe–Ni–N–C (*CO) are the suggested ECR reaction routes on Fe/Ni–N–C. This highlights the increased catalytic activity seen in the studies. The Fe, Ni bimetallic nitrogen-doped carbon successfully lowered the energy barriers of *COOH intermediate production and *CO-to-CO, improving favorable kinetics and increasing ECR activity, verified by the DFT calculations. According to the findings of the calculations above, the suggested ECR reaction pathways of CO_2_ to CO on Fe/Ni–N–C and their energy diagram using DFT calculation are shown in Fig. 1.^84^

## 3. Bimetallic graphene catalysts: mechanistic pathways for CO_2_ reduction and CH_4_ production

Previous studies have shown that single-atom-doped graphene is exceptionally efficient for catalyzing CO_2_RR.^93–96^ Researchers have studied diverse doping techniques for adding transition metals to the graphene, demonstrating that bimetal single-atom-doped catalysts have greater catalytic performance than standard single-atom-doped catalysts.^97–100^ Run Zhang *et al.*, employed DFT calculations for examining CO_2_RR on three bimetal-doped graphene catalysts, Cu–Ni/DG, Cu–Fe/DG, and Fe–Ni/DG. Different reduction pathways yield various products such as CH_4_, CH_3_OH, HCOOH, and CO. In the initial stages of CO_2_RR on doped graphene, CO_2_ adsorption occurs, characterized by analysis of *E*_ads_, electron density difference, density of state (DOS), as well as Integrated Crystal Orbital Hamiltonian Population (ICOHP). Compared to Cu–Fe/DG, CO_2_ reacts more strongly with iron-based Fe–Ni/DG. The catalytic performance of the material is improved by this greater contact. Electron density difference, DOS, and ICOHP studies reveal more robust interactions between certain dopants (Fe and Ni) and CO_2_.^84,101^Table 1 summarizes the adsorption energies of process intermediates on bimetal-doped Fe catalysts. When CO_2_ is first protonated, it produces *COOH or *OCHO, which can then be hydrogenated again to generate CO or HCOOH. These changes proceed in many ways:COOH → *CO + H_2_O → * + CO,andOCHO → *HCOOH → * + HCOOH

**Table 1:** Summary of adsorption energy of different reaction intermediates for the production of CH_4_ on Cu–Fe/DG and Fe–Ni/DG catalysts

Reaction intermediates	Potential energy (eV)	
	Cu–Fe/DG	Fe–Ni/DG
*CO	−1.7	−2.81
*HCOOH	0.78	−0.27
*CH_2_O	−1.46	−2.05
*CH_3_OH	0.97	−0.44
*CH_4_	0.70	−0.76

On Cu–Fe/DG, Cu–Ni/DG, and Fe–Ni/DG catalysts, the high adsorption of CO and HCOOH encourages continued reduction as intermediates. Nevertheless, significant free energy barriers prevent CO and HCOOH from being desorbed from the catalyst's surface, which presents problems for product release. In the CO_2_ reduction reaction, CH_3_OH is a potential product. Four pathways for CO_2_ → CH_3_OH involve *CO or *HCOOH as intermediates. *CO undergoes the following reactions:*CO → *CHO → *CH_2_O (exothermic)or*CHOH → CH_3_OH

*CHO is exothermically converted to *CH_2_O on Cu–Ni/DG and Cu–Fe/DG. On the other hand, *CHO into *CHOH conversions on Fe–Ni/DG are endothermic.*CHO → *CHOH (endothermic)

This distinction draws attention to the different energetics of different catalysts for these reactions.

The thermodynamic favorability of *CH_2_O formation is highlighted by its reduced variance in free energy, which is why it is preferred over *CHOH formation. This preference highlights the role that energetics play in identifying the paths of reactions. Six routes for CO_2_ → CH_4_ reduction were investigated, using *CO or *HCOOH as intermediaries. *CO undergoes the following conversions:*CO → *CHOor*CO → *COH

It resulted in *CHO or *COH and finally CH_4_ by additional hydrogenation. *CO prefers *CHO production because of the lower free energy fluctuation. On Cu–Ni/DG and Cu–Fe/DG,*CHO → *CH_2_O (exothermic)*CHO → *CHOH (endothermic)

While, Fe–Ni/DG prefers,*CHO → *CH_2_O

Path 3 is the best route from Path 1 to Path 5, demonstrating its effectiveness and fit for the intended change. Nevertheless, route 6 (*OCHO → *HCOOH) doesn't work as the best route for Fe–Ni/DG due to significant free energy fluctuation. The optimized pathway is Path 6, which is exothermic on Cu–Fe/DG and Cu–Ni/DG.^101^*CHO → *CH_2_OH → *OCH_3_ → *CH_4_ (exothermic)

Because CO_2_ interacts with Fe or Ni atoms more strongly than its interaction with Cu, Fe–Ni/DG is more stable than Cu–Ni/DG and Cu–Fe/DG. The catalytic potential of Fe–Ni/DG for CO_2_ conversion reactions is highlighted by its improved stability. Fe–Ni/DG becomes more prominent in bimetal-doped graphene systems because of its increased stability, which implies that it can support effective and long-lasting catalytic activity. Graphene doped with Cu, Fe, and Ni shows significant selectivity for CO_2_ reduction over hydrogen evolution (HER), suggesting that these materials are effective catalysts for CO_2_ conversion processes, with various product outcomes seen for the initial protonation step of CO_2_ on these catalysts.

## 4. FeCo-Pc catalysts for multi-carbon (C_2_) product formation

FeCo-Pc catalyst with dual metal–nitrogen active sites for efficient CO_2_RR. FeCo-Pc overcomes the challenge of C–C coupling seen in single-atom catalysts,^102,103^ enabling the production of C_2_ products, as shown in Table 2. These C_2_ products include C_2_H_4_, CH_2_OHCH_2_OH, C_2_H_5_OH, and CH_3_COOH with enhanced selectivity, due to the cumulative effects of Fe and Co dual active sites anchored within phthalocyanine (FeCo-Pc).^104^ The computations are performed using the Gaussian 09 program, PBE exchange–correlation functional, and 6-31G* basis sets.^105,106^ C–C coupling processes in CO_2_ reduction are critical for comprehending multi-carbon product generation.^107,108^ Water plays an important role in the reduction of CO_2_ because it influences intermediate hydration, provides protons for product production, and functions as a solvent for ion transport. Additionally, through the oxygen evolution reaction (OER), it competes with CO_2_ reduction and affects catalyst behavior.^109,110^ CO was identified as a crucial intermediate, discovered by *in situ* spectroscopy.^111,112^ PCETs generate C_1_ intermediates like CHO* and COH*, with thermodynamics and kinetics assessed on FeCo-Pc surfaces. CO to CHO* (formyl group) is produced by further reducing *CO and adding an extra proton and electron. Usually, this process produces more valuable chemicals such as alcohols and aldehydes. In the case of COH* protonation step is required for the formation of COH*, but not the complete reduction required to generate *CHO. In the synthesis of other C_1_ products, such as ethanol or methane, this is frequently a transitional stage. CO dimerization, proposed as the initial step to C_2+_ products, and carbene 
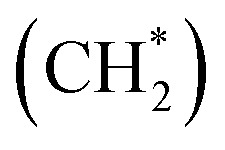
 coupling with CO* to form 
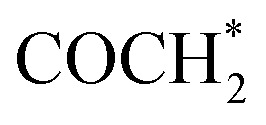
 or CH_2_CO*, are investigated.^42,113^ However, the generation of “dead-end” intermediates, such as COCO* and other C–C linked intermediates, tightly bound to FeCo-Pc, incurs high energy consumption, creating kinetic obstacles and surface contamination. Electrolytes C–C coupling pathways are influenced by pH,^114^ with high pH favoring CO* to COH* conversion and CO* coupling with CHO* to generate COCHO*, boosting C_2_ synthesis.^115,116^ COCHO* formation has a lower activation barrier compared to CHO*–CO* precursors, demonstrating its thermodynamic favorability and potential to provide more C_2_ products in CO_2_RR on FeCo-Pc surfaces.^117^ In an aqueous solution, the relative stability of COOH* as well as OCHO* in CO_2_RR against H* in HER affects the competing processes of the H_2_ evolution reaction (HER).^118^ FeCo-Pc prefers CO_2_RR over HER due to larger free energy changes for COOH* and OCHO* production against adsorbed H*. COOH* has a smaller overpotential than OCHO*, indicating FeCo-Pc prefers CO_2_RR.^119,120^ The electro-conversion of CO_2_ to C_2_H_4_ is important for the C_2_H_4_ industry, however, it faces challenges with an elevated overpotential and multi-electron transfer processes.^121,122^

**Table 2:** Summary of reaction intermediates generates during the formation of C_2+_ products with their possible potential energy over FeCo-Pc catalyst

Compound	Reaction intermediate	Potential energy (eV)
C_2_H_4_	COOH*	0.13
	CO*	0.23
	CHO*	0.68
	COCHO*	0.70
	CHOCHO*	0.38
	CHOHCHO*	0.55
	CHCHO*	0.27
	CH_2_CHO*	0.17
	CH_2_CHOH*	0.06
	CH_2_CH*	0.51
C_2_H_5_OH	CHOHCH_2_O*	0.41
	CHCH_2_O*	0.21
	CH_2_CH_2_O*	0.40
	C_2_H_2_OH*	0.71
CH_3_COOH	COCHOH*	0.57
	COCH_2_O*	1.59
	COHCH_2_O*	0.26
	CH_2_COOH*	0.60
CH_2_OHCH_2_OH	CHOHCH_2_OH*	0.16
	CH_2_OHCH_2_OH*	0.20

FeCo-Pc catalysts increase CO_2_RR by favoring COOH* over H*, resulting in C_2_H_4_ generation. During CO reduction, CHO* formation takes precedence over COH* formation. The rate-limiting step for C_2_H_4_ generation is coupling CHO* with CO* to generate COCHO*.^22^ FeCo-Pc surfaces aid in producing C_2_H_4_ by reducing COCHO* to glycolaldehyde and hydrogenating further. FeCo-Pc catalysts provide a viable avenue for the electrochemical process to transform CO_2_ into ethanol (C_2_H_5_OH), a critical commodity chemical, *via* C–C coupling reactions. The procedure is optimized by hydrogenating typical intermediates with ethylene (C_2_H_4_). CHOHCHO* is found as a selectivity-determining molecule. Thermodynamically, C_2_H_5_OH is produced through the optimum process of CHOHCHO* hydrogenation to CHOHCH_2_O*.^117,123,124^

**Scheme 1:**
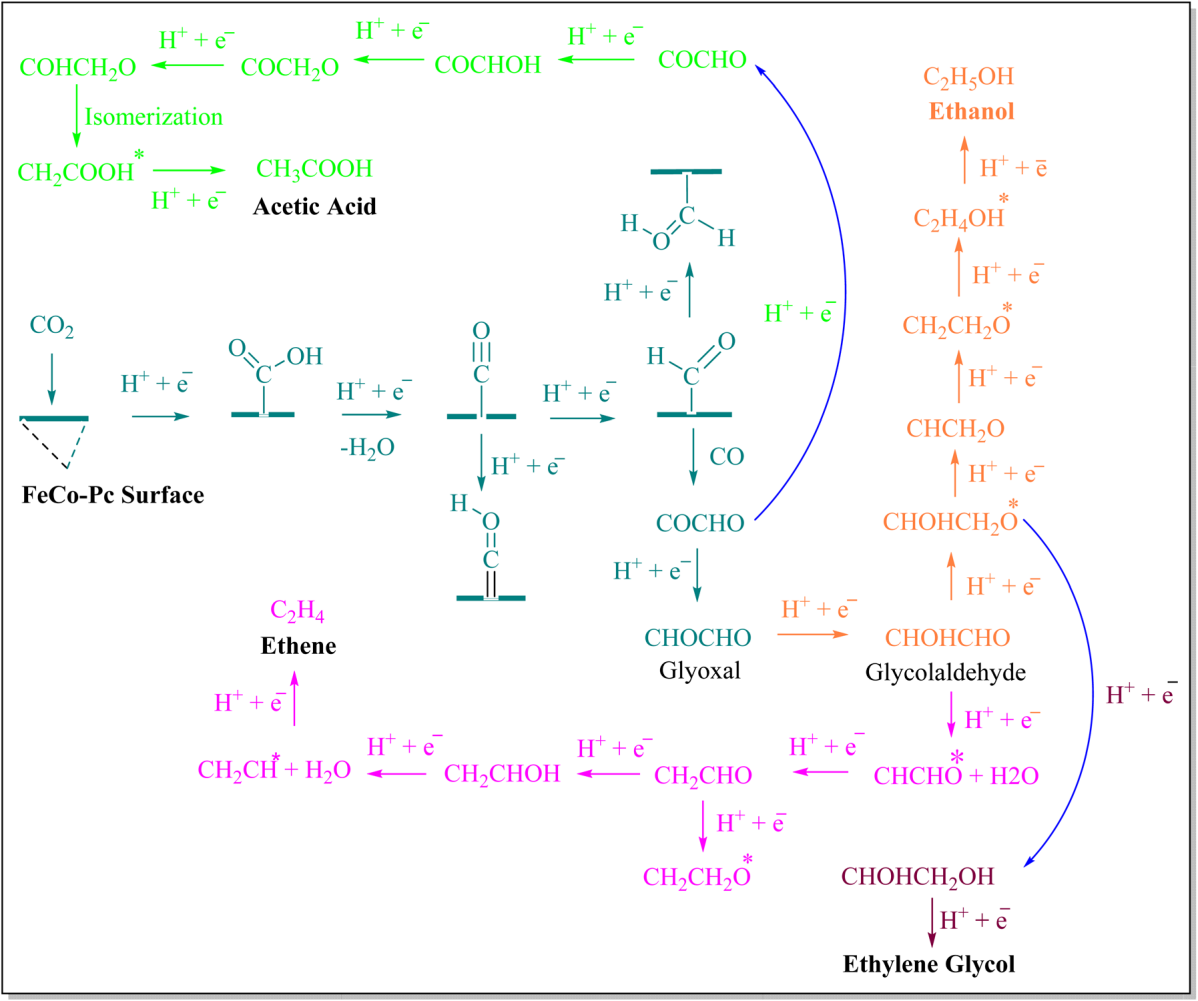
Possible electrochemical reaction pathways of CO_2_ over Fe-based bimetallic catalyst into C_2+_ products.

The rate-limiting step (RLS) for C_2_H_5_OH creation involves the hydrogenation process of CH_2_CH_2_O* to C_2_H_4_OH*, which has a greater barrier than the formation of C_2_H_4_. The preference for CHCHO* or CHOHCH_2_O* production during CHOHCHO* reduction determines the selectivity of C_2_H_4_ and C_2_H_5_OH. Increasing potential increases the feasibility of producing C_2_H_4_ and C_2_H_5_OH on FeCo-Pc, with all fundamental stages downward energetically at −0.66 V-RHE. Previous investigations have discovered ethylene glycol to be a negligible product in CO_2_RR utilizing catalysts such as Au, Ru, and Cu.^125–127^ Calvinho *et al.*, recently proved that CO_2_RR may be converted to ethylene glycol (CH_2_OHCH_2_OH) using a transition-metal phosphide catalyst. CHOHCHO*, like C_2_H_4_ and C_2_H_5_OH, determines the selectivity of CH_2_OHCH_2_OH production. Protonation of CHOHCH_2_O* results in the formation of CHOHCH_2_OH*, which is preferred over CHCH_2_O*. This is then transformed into ethylene glycol. Geometry optimization demonstrates that CH_2_OH is not chemisorbed, indicating that it prefers the formation pathway over C_2_H_5_OH. Both CH_2_OHCH_2_OH and C_2_H_5_OH have an identical kinetic barrier for CHOHCH_2_O* production, resulting in C_2_H_4_ selectivity.^104,128–130^ Possible electrochemical reduction pathways for CO_2_ into C_2_ products are shown in Scheme 1.

## 5. Fe-based bimetallic electrocatalysts: advanced pathways for CO_2_ reduction

**Fig. 2:**
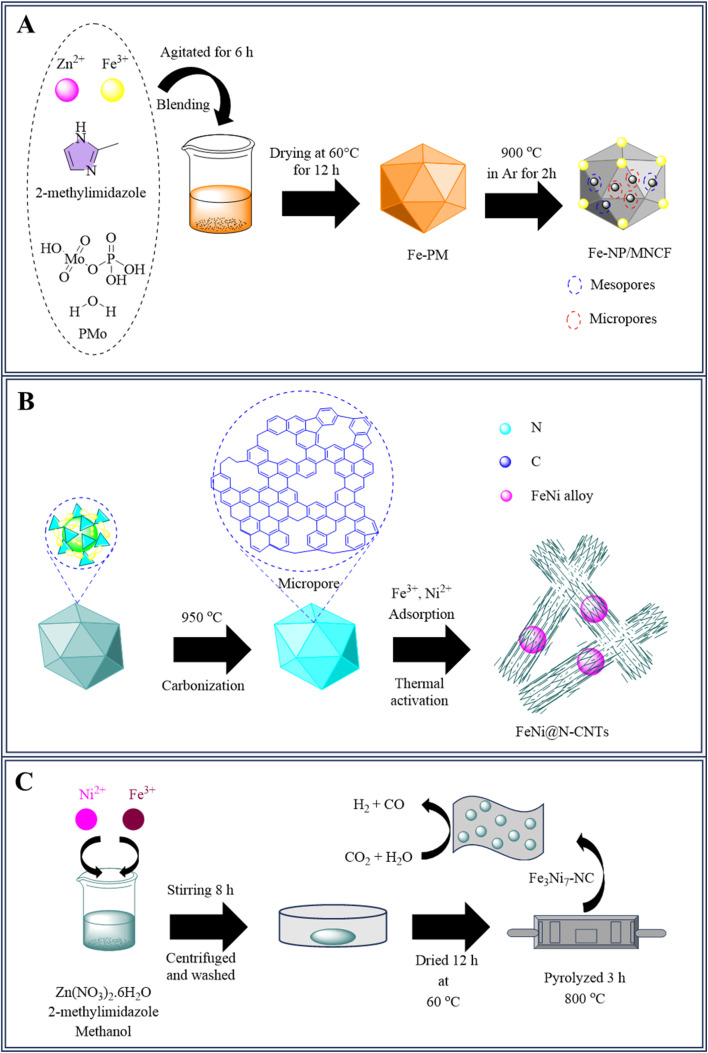
(A) Synthesis of Fe-NP/MNCF (B) synthesis of FeNi@N-CNTs and (C) synthesis of Fe_3_Ni_7_–N–C.

As previously reported, nitrogen-doped carbon nanotubes with Fe/Fe_3_N nanoparticles improve the catalytic performance of the oxygen reduction process (ORR) by exposing active areas and enabling electron transport.^131–134^ Before pyrolysis, Fe-doped zinc-imidazole frameworks (ZIF-8) were changed with phosphomolybdic acid hydrate (PMo), resulting in the formation of Fe nanoparticles contained within molybdenum and nitrogen-co-doped carbon scaffolds (Fe-NP/MNCF). In CO_2_ electrolysis powered by a Zn–air battery (ZAB), Fe-NP/MNCF served as a dual-functional catalyst during ORR and CO_2_RR. To synthesize, Fe(NO_3_)_3_·9H_2_O, Zn(NO_3_)_2_·6H_2_O, and PMo were dispersed in 2.5 mL of water that was deionized by ultrasound. The precursor was calcined at 900 °C in an argon environment for 2 hours to produce Fe-NP/MNCF, as illustrated in Fig. 2A.^135,136^

Nitrogen-doped carbon was obtained by annealing ZIF-8 at 950 °C in an argon environment. The final product, called ZIF-NC, has a three-dimensional porous structure.^137^ FeNi@N-CNTs catalysts were developed by wet impregnation and thermal processing. The heat was applied at 1100 °C to produce FeNi@N-CNTs-*X*, where *X* is the temperature at which they were annealed (Fig. 2B). By encasing the FeNi alloy in N-CNTs, this synthesis method enhances the catalytic properties for potential CO_2_ reduction uses. Better CO_2_ER activity and stability were demonstrated by FeNi@N-CNTs-1100, which showed over 90% CO faradaic efficiency spanning a wide potential range (−0.47 to −0.97 V *vs.* RHE). Optimized *COOH adsorption and *CO desorption were credited with improving catalytic activity and CO selectivity.^138,139^

Xiao Han *et al.* employed a solution approach to create FeNi precursors, which were subsequently transformed into a variety of FeNi-NC catalysts *via* one-step pyrolysis. Dissolved Ni(NO_3_)_2_·6H_2_O, Zn(NO_3_)_2_·6H_2_O, and Fe(NO_3_)_3_·9H_2_O in 80 mL of methanol and stirred thoroughly. Separately, another 80 mL of methanol was used to dissolve 2-methylimidazole and added to the metal nitrate solution. The resultant mixture was agitated constantly for 8 hours to produce the catalyst precipitate. The resulting precipitate washed away with the solvent methanol centrifuged, evaporated at 60 °C, and powdered to produce Fe_3_Ni_7_-ZIF samples with different Fe/Ni ratios, as illustrated in Fig. 2C. These catalysts attain about 100% overall Faraday efficiency by promoting CO_2_ electroreduction into CO and H_2_. Furthermore, we discovered that altering the applied potential across a large range throughout the procedure makes it simple to change the syngas ratio from 1 : 1 to 6 : 1 (CO/H_2_). Because of its versatility, syngas can be utilized to manufacture fuels and raw materials for chemicals.^140^

The unique features of doped Cu in Fe–N–C catalysts, including its numerous oxidation states, which facilitate fast electron transfer,^87,141^ ability to particularly manufacture C_2+_ products,^142–144^ and improved interaction with CO_2_ to limit hydrogen development, have sparked great interest in CO_2_RR. The Fe/Cu–N–C catalyst, which was produced by adding a copper promoter to a mixture of iron and carbon sources and then pyrolyzing it, has outstanding CO_2_ reduction efficiency with more than 90% CO faradaic productivity (FE_CO_) in a broad potential range (−0.5 to −0.7 V) and remarkable stability, with FE_CO_ maintained after 10 hours of electrolysis. To make the Fe/Cu–N–C catalyst, Shulin Zhao *et al.*, mixed tris(2,4-pentanedionato)iron(iii), Cu-acetylacetonate, along with meso-tetra(4-methoxyphenyl) porphin in CHCl_3_ and stirred it at 60 °C for the period of 3 h. Rotational evaporation was used to extract the solvent from the mixture after 30 minutes of sonication following the addition of zinc oxide. After the powder was produced, it was heated to 900 °C in an argon atmosphere for two hours, then it was leached for six hours at 80 °C in 0.5 M H_2_SO_4_ and allowed to dry overnight.^145^Table 3 shows the comparative analysis of heteronuclear Fe-based catalysts for CO_2_ electroreduction.

**Table 3:** Comparative analysis of heteronuclear Fe-based catalysts for CO_2_ electroreduction

Catalyst	Synthesis method	Key features	CO_2_ reduction efficiency	Stability	Reference
Fe-NP/MNCF	Fe-doped ZIF-8 modified with PMo, pyrolysis at 900 °C	Molybdenum and nitrogen co-doped carbon scaffold	The dual-functional catalyst for ORR and CO_2_RR	Used in Zn–air battery-powered CO_2_ electrolysis	135 and 136
ZIF-NC	Annealing ZIF-8 at 950 °C	Three-dimensional porous structure	—	—	137
FeNi@N-CNTs-*X*	Wet impregnation and thermal processing at 1100 °C	Encapsulated FeNi alloy in N-CNTs, enhanced CO_2_ reduction	>90% CO faradaic efficiency (−0.47 to −0.97 V *vs.* RHE)	High stability	138 and 139
Fe_3_Ni_7_-ZIF	Solution approach, pyrolysis	Various Fe/Ni ratios to adjust performance	∼100% faraday efficiency, tunable syngas ratio (1 : 1 to 6 : 1 CO/H_2_)	High stability, broad potential range	140
Fe/Cu–N–C	Cu promoter added to Fe/carbon mixture, pyrolysis at 900 °C, acid leaching	Enhanced electron transfer, improved CO_2_ adsorption	>90% CO faradaic efficiency (−0.5 to −0.7 V)	Stable after 10 hours of electrolysis	145
C–Fe–Co-ZIF	Impregnation of ZIF-8 with Fe and Co, pyrolysis	Bimetallic Co–Fe catalyst for CO_2_ electroreduction	+10% CO faradaic efficiency *vs.* pure Co-ZIF	H_2_/CO ratios tunable (0.8 to 4.2), 93% FE_CO_ + H_2_ over 10 hours	146
Fe/Mn–N–C	Potassium citrate calcination, Fe and Mn doping, pyrolysis at 800 °C	Atomic dispersion of Fe and Mn for CO selectivity	94% CO faradaic efficiency at −0.5 V (RHE)	>80% FE_CO_ after 12 hours	146 and 147

**Fig. 3:**
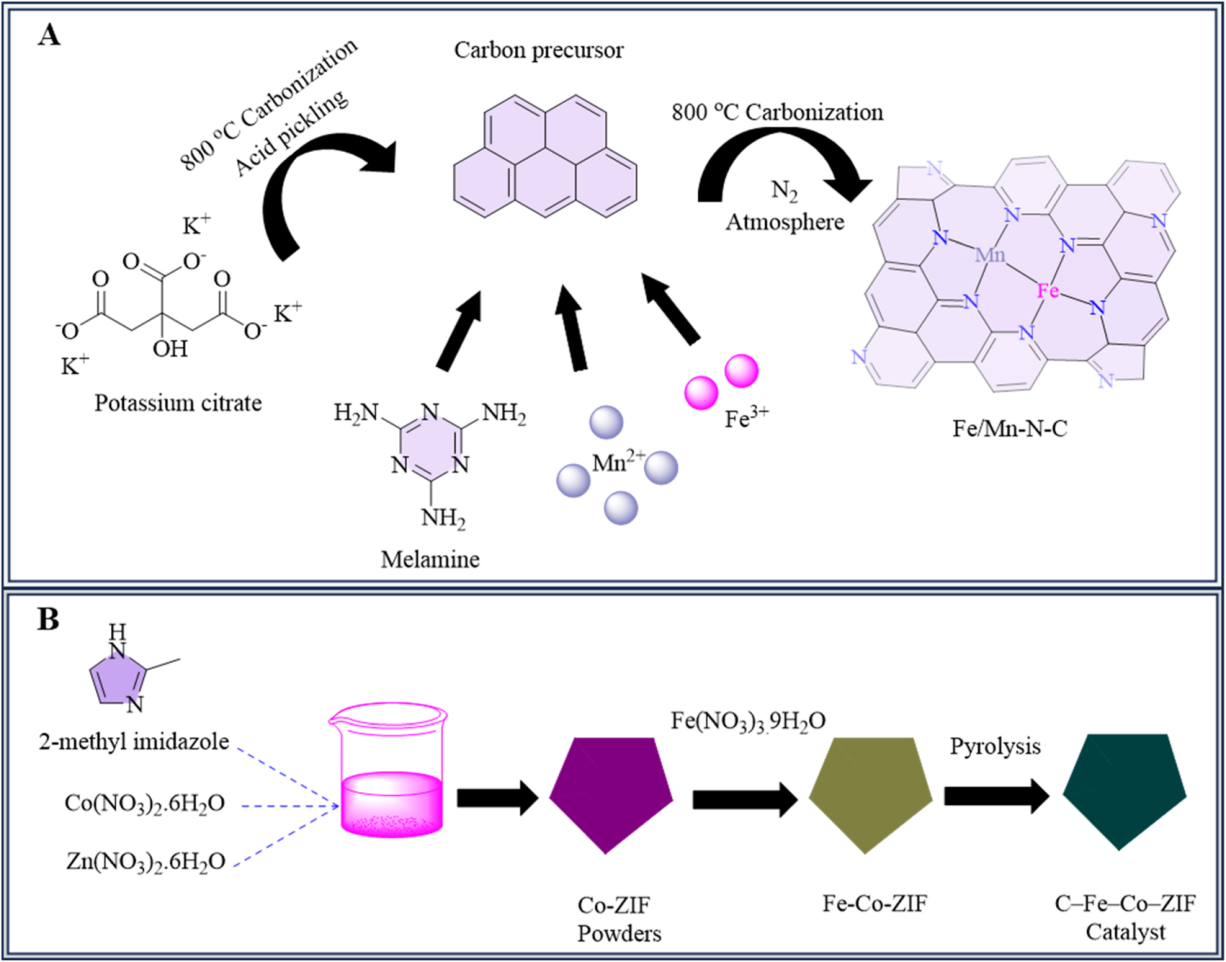
Methodology for the synthesis of (A) Fe/Mn–N–C and (B) C–Fe–Co-ZIF catalysts.

The production of Fe/Mn–N–C, a unique bimetallic catalyst consisting of iron and manganese atomic dispersion, involved the elevated temperatures calcination of an organic carbon-based porous precursor. The solution of potassium citrate monohydrate was initially calcined for an hour at 800 °C in a nitrogen atmosphere to create porous black carbon compounds. The resulting solid was dried in the oven for 12 hours at 80 °C after being rinsed with deionized water and a 1 M H_2_SO_4_ solution until it attained a neutral pH. A mixture consisting of carbon material, Fe(NO_3_)_3_·9H_2_O, and MnCl_2_·4H_2_O in deionized H_2_O was ultrasonically treated for an hour, centrifuged, and dried afterward. The resultant solid was combined with melamine in a particular mass ratio and then calcined at 800 °C, over a nitrogen environment for two hours to generate the Fe/Mn–N–C catalyst,^146^Fig. 3A. At a −0.5 V overpotential (RHE), the Fe/Mn–N–C catalyst produced a 94% Faraday efficiency (FE) for CO in the 0.1 M KHCO_3_ electrolyte. This shows that, in these electrochemical circumstances, the catalyst has a high selectivity for CO synthesis. The catalyst's performance is notable when compared to previously published iron-based and manganese-based electrocatalysts, which include FeMn–N–C (FE_CO_ 80% at −0.5 V RHE), NFe-CNT/CNS (FE_CO_ 69% at −0.6 V RHE), and Mn–N–C (FE_CO_ 70% at −0.6 V RHE).^53,148,149^ Following just 12 hours of uninterrupted catalysis, the FE_CO_ was above 80%, suggesting good stability. Density functional theory (DFT) calculations show that the interaction of neighboring Fe–Mn centers lowers the potential for COOH* production and CO desorption.^146^

## 6. Development of atomically distributed Co–Fe catalysts for CO_2_ reduction

Bimetallic Co–Fe catalysts that are atomically distributed were developed in two steps. Using this method, the catalysts were synthesized with accurate atomic-level dispersion of iron and cobalt through a series of synthesis steps. To ensure a successful yield without interference in the crystallization of Co-ZIF, Fe–Co-ZIF precursors were generated by an impregnation process that modified ZIF-8 into Co-ZIF and absorbed Fe source. Pyrolysis was then used to manufacture the final catalysts (C–Fe–Co-ZIF) for CO_2_ electro-reduction,^150^ shown in Fig. 3B. The bimetallic catalysts produced more CO, with an additional 10% in CO Faradaic efficiency (FE) when compared to pure C–Co-ZIF. Adjustable H_2_/CO ratios (0.8 to 4.2) reached across a wide potential range, with a high overall FE CO + H_2_ of 93% over 10 hours, showing the catalyst's capacity for efficient syngas production from CO_2_.^147^

## 7. Graphene oxide-based catalysts for CO_2_ reduction

Graphene oxide (GO) was produced with graphite using the modified Hummers' method.^151^ GO suspension (2 mg mL^−1^) was made by sonicating it in deionized water for 5 hours. Iron and nickel nitrates were introduced to the GO solution, which was sonicated for three more hours. The resulting slurry was heated to 180 °C in an autoclave lined with Teflon for 12 h before being freeze-dried to generate a columnar product. Subsequently, a chemical vapor deposition (CVD) process at 1000 °C with Ar and NH_3_ was used to synthesize the H–NiFe/NG composite, followed by annealing with hot steam. A novel method involving steam-assisted chemical vapor deposition introduces surface oxygen vacancies (V_O_) into Ni–Fe BM NPs, creating electron-rich centers that activate CO_2_ molecules.^152,153^ This method reduces the energy barrier for creating COOH* intermediates, increasing the reduction of carbon dioxide to CO while maintaining a faradaic efficiency of as high as 94% at −0.80 V (*vs.* RHE) along with excellent stability. Surface V_O_-modified atoms of nickel have a vital role in increasing the electrocatalytic efficacy of reduction of CO_2_ to CO, according to density functional theory simulations.^154^

## 8. Molecular catalyst-based heterostructures for CO_2_ reduction

The design and synthesis of a molecular catalyst-based heterostructure for the reduction of CO_2_ is still a serious issue. Molecular catalysts with transition-metal elements (Co, Ru, Fe, Ni, Cu) and ligands made of organic compounds (phthalocyanine, polypyridine, porphyrin) provide precise active sites and structural tunability for researching CO_2_ER processes.^155–159^ These catalysts facilitate detailed investigations into CO_2_ reduction catalysis. A crystalline bimetallic phthalocyanine heterostructure electrocatalyst (CoPc/FePc HS) was developed for CO_2_ reduction, achieving a remarkable CO_2_ to CO conversion efficiency of 99% at the potential of −0.87 *vs.* RHE and demonstrating outstanding stability over 10 h of electrocatalysis. Different Co/Fe molar ratios (3 : 1, 1 : 1, 1 : 3)^160^ of CoPc/FePc heterostructures, along with CoPc and FePc controls were synthesized by dispersing a mixture of CoPc and FePc in DMF and subjecting it to solvothermal treatment at 180 °C for 24 hours. Precipitates in the shape of purple microrods were gathered and cleaned with ethanol. They were then calcined for three hours at 450 °C in an Ar environment. CoPc/FePc heterostructures were formed as a consequence of this technique. This method provides a controlled approach to tailor the composition of bimetallic phthalocyanine heterostructures for CO_2_ reduction applications.^161^

## 9. Cu–Fe–N_6_–C: a high-performance diatomic site catalyst for CO_2_ reduction

**Fig. 4:**
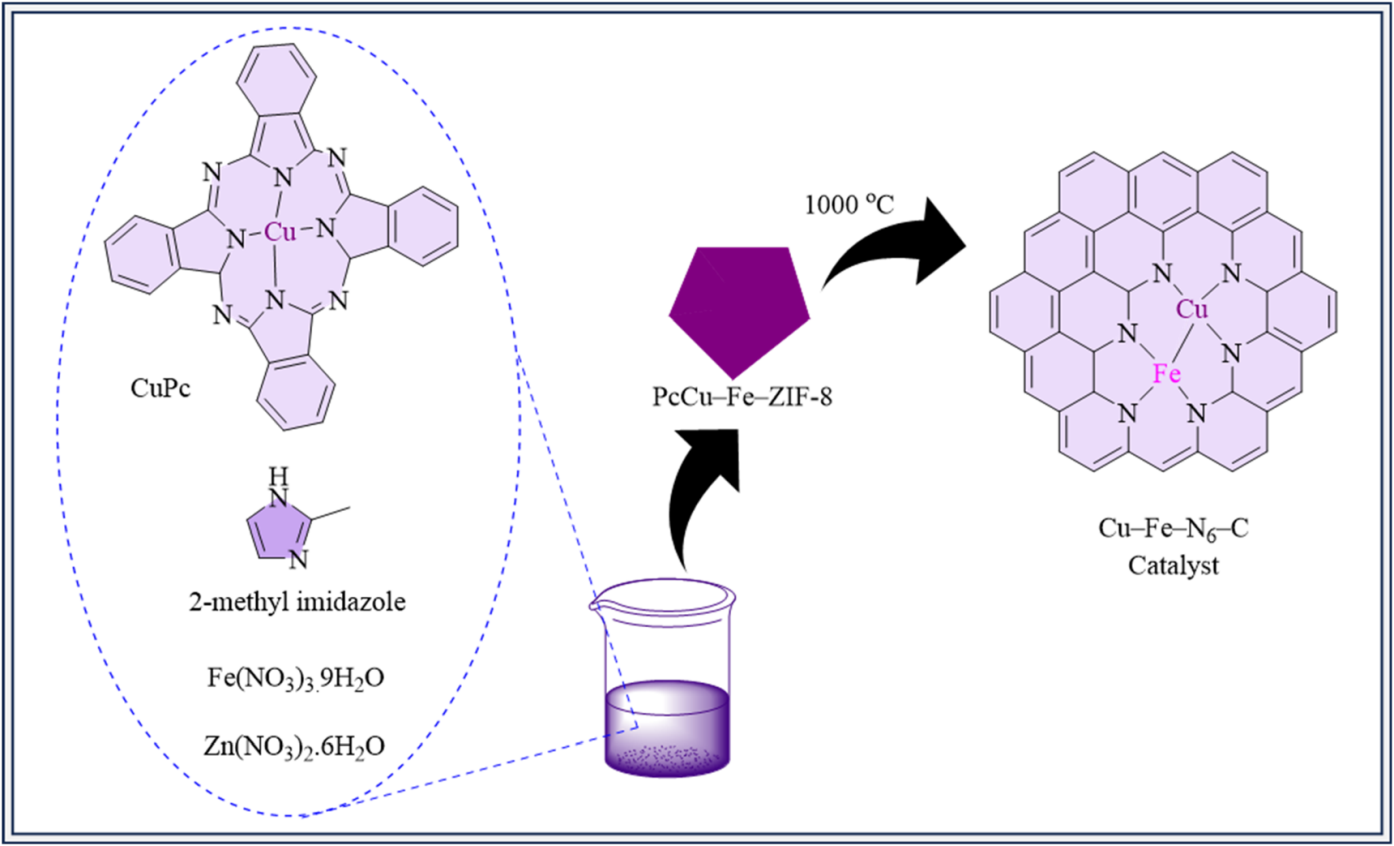
Synthesis of Cu–Fe–N_6_–C.

Metal–nitrogen–carbon (M–N–C) catalysts have great potential for CO_2_ electrocatalytic reduction because of their abundance of active sites and low-cost raw ingredients.^52,162–165^ Cu–Fe–N_6_–C, a new diatomic site catalyst coordinated with nitrogen and embedded into a carbon matrix, was developed, and synthesized. Cu–Fe–N_6_–C was synthesized in two primary stages. First, PcCu-Fe-ZIF-8 is created by combining PcCu, zinc nitrate, iron nitrate, and 2-Me–imidazole, resulting in a blue precipitate that indicates uniform dispersion of Cu and Fe species inside the framework. PcCu-Fe-ZIF-8 becomes Cu–Fe bimetallic sites distributed on a nitrogen-doped carbon framework upon annealing at 1000 °C under Ar. The necessity for extra acid leaching treatment is eliminated by this technique. For a variety of processes, the resulting catalyst structure improves catalytic performance (Fig. 4). This catalyst outperformed individual Cu–N–C and Fe–N–C catalysts thanks to synergistic effects at bimetallic sites. Cu–Fe–N_6_–C demonstrated outstanding CO selectivity, with an exceptional faradaic efficiency of 98% at −0.7 V, and maintained selectivity after 10 hours of electrolysis. Experimental and theoretical investigations revealed that the combined catalysis of several metallic sites increased CO_2_ adsorption enthalpy, and lowered activation energy, resulting in enhanced selectivity, activity, and stability, as well as decreased impedance in CO_2_ hydrogenation.^166^

For CO_2_ conversion, some Na-promoted Co–Fe bimetallic catalysts ranging in proximity and compositions were investigated. These catalysts are designed to use the strong selectivity of iron for olefins during CO_2_ hydrogenation, along with the high activity and reducibility of cobalt. The goal of this combination is to improve CO_2_ conversion operations' overall efficiency and selectivity.^167–170^ Co-precipitation was used to produce Co–Fe bimetallic catalysts, which were then hydrothermally treated. The manufacture of uniform catalysts with regulated compositions and architectures is made easier by this technique.^171,172^ Co(NO_3_)_2_·6H_2_O and Fe(NO_3_)_3_·9H_2_O were dissolved sequentially in deionized water to achieve a [Co]^2+^ + [Fe]^3+^ concentration of 0.09 M, followed by the addition of 5 mol L^−1^ NaOH solution until pH 11 was reached. The resultant hydroxide precipitates were hydrothermally treated at 150 °C for 24 hours before being centrifuged, washed, and dried at 80 °C. The dried products were calcined at 400 °C for 3 hours to produce Co–Fe catalysts with various Co/Fe molar ratios (1/4, 1/2, 1/1, 2/1, and 4/1), designated as Co1Fe4, Co1Fe2, Co1Fe1, Co2Fe1, and Co4Fe1, respectively. The Co1Fe2 catalyst, having a Co/Fe molar proportion of 1/2 and proximity, permitted the quick reduction of CoFe_2_O_4_ to Co_*x*_Fe_*y*_ alloy and subsequently carbonization to *χ*-(Co_*x*_Fe_1−*x*_)_5_C_2_ alloy carbide. It demonstrated improved stability and performance in olefin production without deactivation over 500 h on-stream.^173^

## 10. Fe/Ni–N–C catalysts with 3D carbon-based structures for CO_2_ reduction

**Fig. 5:**
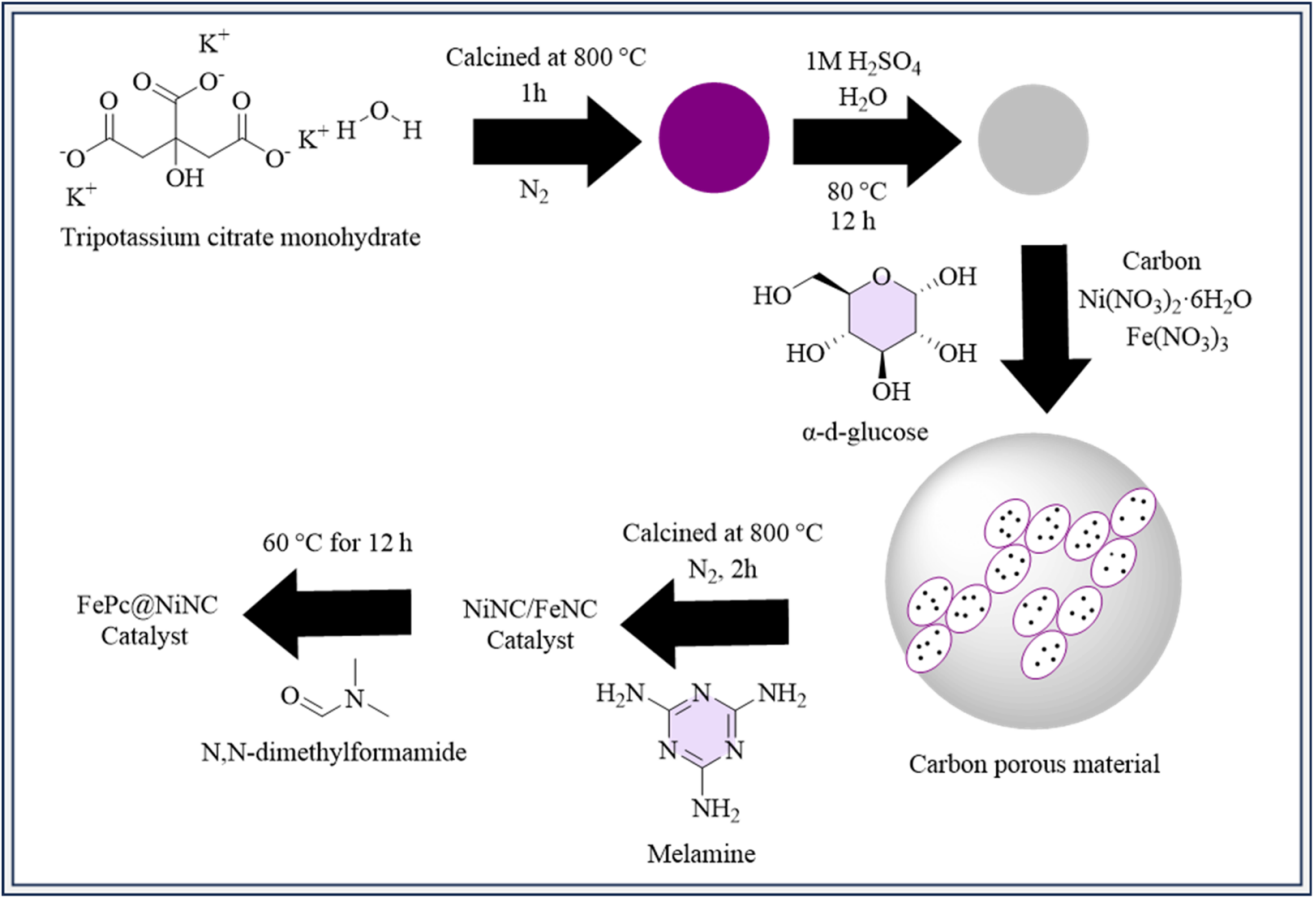
Methodology for the synthesis of FePc@NiNC catalyst.

A 3D carbon-based material was produced, featuring bimetallic centers^174^ that include NiNC and FeNC, which demonstrated synergistic effects advantageous to the CO_2_RR. The synthesis procedure involved numerous steps to produce various catalyst materials. Tripotassium citrate monohydrate was cooked at 800 °C under nitrogen, and then treated with sulfuric acid and water to create a porous carbon material. Next, a mixture containing carbon, nickel nitrate, iron nitrate, and glucose in water was processed using ultrasound and then combined with melamine. This mixture was heated at 800 °C under nitrogen to produce the NiNC/FeNC catalyst.^175^ Further, specific catalysts like FePc@NiNC and NiPc@FeNC were prepared by treating NiNC or FeNC with *N*,*N*-dimethylformamide and adding iron phthalocyanine (FePc) or nickel phthalocyanine (NiPc), respectively (Fig. 5). Each stage required precise chemical reactions and thermal treatments to generate the correct catalyst compositions. DFT models and observations show^176^ Fe atoms are reactive and adsorption sites for CO_2_RR, while substantial CO* adsorption reduces stability. By adding Ni atoms, CO* adsorption on Fe is decreased, changing the energy barriers and improving stability. The Fe–N_4_ and Ni–N_4_ sites work in concert to facilitate the rate-limiting processes (CO_2(g)_ → COOH*, +0.95 eV) in FePc@NiNC. Flexible syngas composition is made possible by this synergy while high catalytic activity is maintained.^177^

## 11. Ni/Fe–N–C: diatomic metal–nitrogen catalysts for CO_2_ reduction

**Fig. 6:**
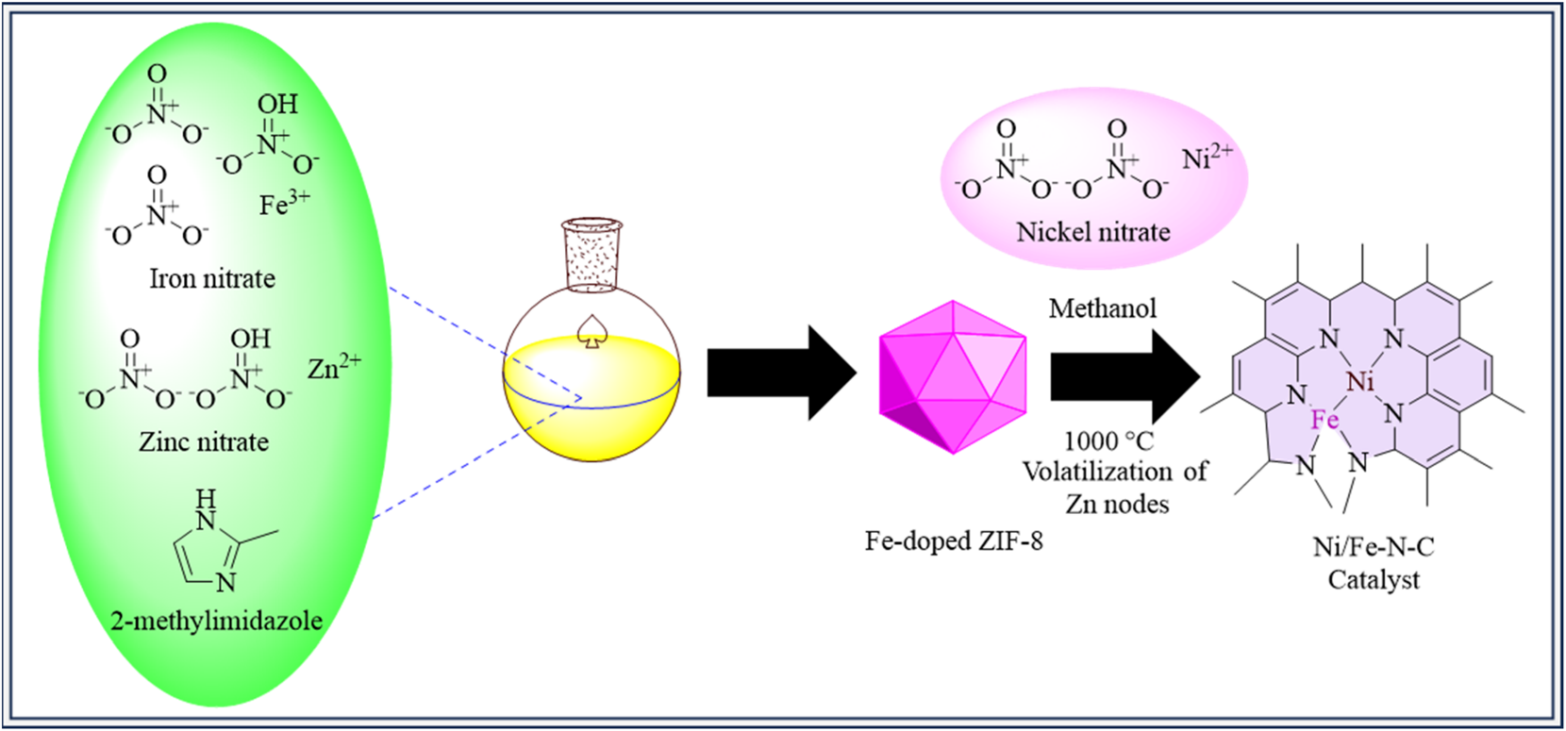
Methodology for the synthesis of Ni/Fe–N–C catalyst.

A ZIF-8 was used to create a catalyst consisting of isolated diatomic metal–nitrogen species. Initially, Fe-doped ZIF-8 was made by combining zinc nitrate, iron nitrate, and 2-methylimidazole, maintaining that Fe ions were chemically bound to the organic ligand rather than being physically absorbed.^178^ Fe-doped ZIF-8 was dissolved in *n*-hexane, and nickel nitrate methanol solution was added gradually. Nickel was contained within ZIF-8's tiny cavities using this method. Nickel was well incorporated into the framework owing to the steady infusion.^90,179^ After thermal treatment at 1000 °C, the resulting catalyst, Ni/Fe–N–C, containing nitrogen-coordinated diatomic Ni–Fe species, was obtained (Fig. 6). For comparison, crystalline Ni–N–C and Fe–N–C catalysts were synthesized similarly. After 30 hours, the Ni/Fe–N–C catalyst retains 99% selectivity and over 90% CO faradaic efficiency from −0.5 to −0.9 V, which ended at 98% at −0.7 V. Synergistic Ni–Fe interactions lower CO_2_ reduction reaction barriers and cause structural changes upon CO_2_ adsorption, according to DFT research, improving the catalyst's performance.^180^

## 12. Micelle-encapsulated Fe-based nanoparticles for CO_2_ reduction

**Fig. 7:**
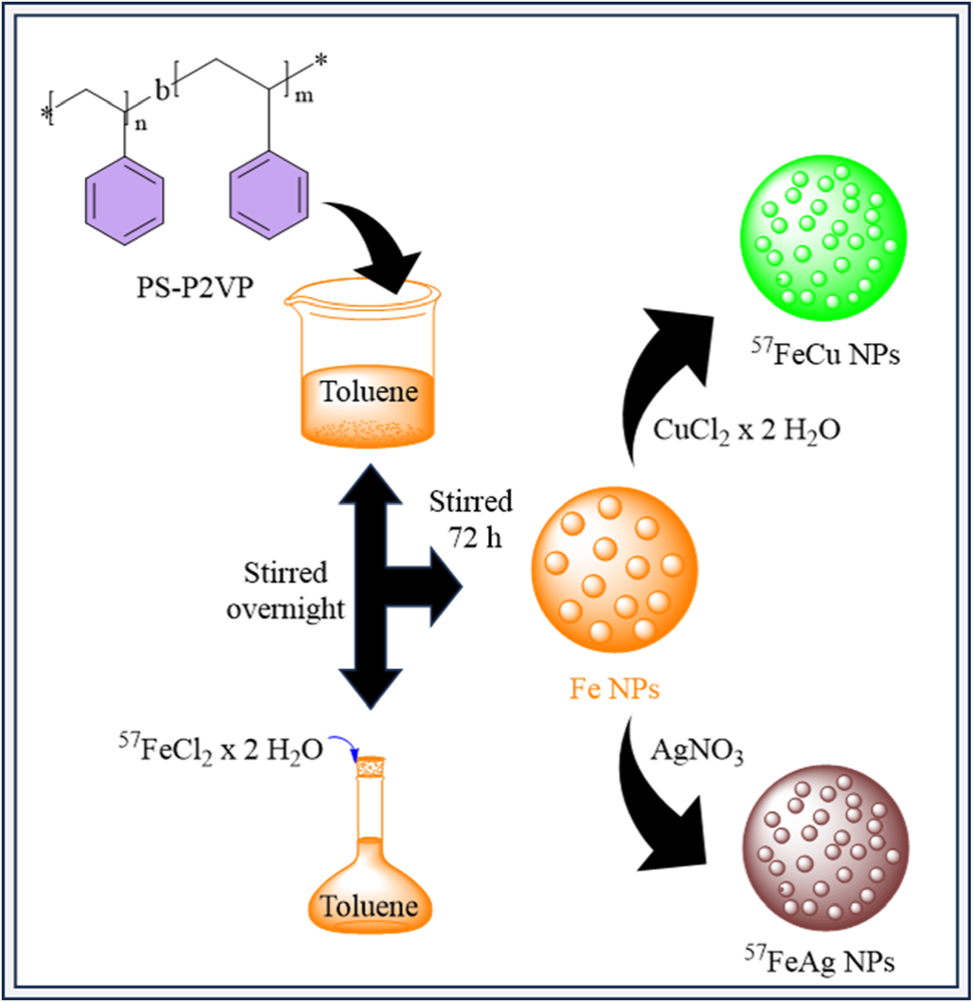
Synthesis of ^57^FeCu and ^57^FeAg NPs for CO_2_ERR.

Inverse micelle encapsulation was used to produce size-selected nanoparticles (NPs) of Fe, FeCu, FeAg, Ag, and Cu. The poly(styrene)-*block*-poly(2-vinylpyridine) (PS-P2VP) diblock copolymer, which was obtained from Polymer Source Inc., was used in this procedure. Metallic salts (FeCl_2_, AgNO_3_, CuCl_2_, FeCl_3_) and the copolymer were dissolved in toluene. By taking advantage of the micelles encapsulating attributes, this technique made controlled nanoparticle manufacturing easier.^181,182^ Specifically, isotopically enriched ^57^FeCl_2_ salt was employed for NRIXS measurements, prepared from iron foil with 95% ^57^Fe isotopic enrichment using adapted literature procedures.^183^ Following NP synthesis, the samples were soaked with carbon black powder and then treated using N_2_-plasma to eliminate the polymer, resulting in clean NP surfaces. The NPs were subsequently distributed into an ethanol/Nafion solution enabling electrode deposition, accompanied by further N_2_-plasma treatment to remove any remaining polymer before electrochemical evaluation. Fig. 7 shows that the production of ^57^Fe NPs involves mixing PS-P2VP in toluene to create reverse micelles, which were then added to ^57^FeCl_2_ salt and stirred for 72 h. Similar methods were utilized to create ^57^FeCu and ^57^FeAg NPs by changing the ratios of ^57^FeCl_2_ to CuCl_2_ or AgNO_3_ in the micellar solution. FeAg NPs had 36% CO faradaic selectivity at −1.1 V *vs.* RHE in 0.1 M KHCO_3_, similar to pure Ag NPs, but FeCu NPs prefer H_2_ evolution, similar to pure Fe NPs.^184^Table 4 summary of recently reported Fe-based bimetallic electrocatalysts for CO_2_ reduction.

**Table 4:** Summary of recently reported Fe-based bimetallic electrocatalysts for CO_2_ reduction

Catalyst	Electrolyte	Major product	FE (%)	Potential (V)	Current density (mA cm^−2^)	Stability
Fe-NP/MNCF	0.5 M KHCO_3_	CO, H_2_	87.50%	−0.7	10	36 h
FeNi@N-CNTs	0.5 M KHCO_3_	CO	90%	−0.47 to −0.97	20.18	35 h
Fe_3_Ni_7_-ZIF	0.5 M KHCO_3_	CO, H_2_	81.30%	−0.9	−22.5	Good
Fe/Cu–N–C	0.5 M KHCO_3_	CO	97%	−0.6	74	10 h
Fe/Mn–N–C	0.1 M KHCO_3_	CO	94%	−0.5	−83.5	12 h
Fe/Ni-ZIF-8	0.5 M KHCO_3_	CO	89%	−0.677	26.92	40 h
FePc@NiNC	0.5 M KHCO_3_	CO, H_2_	100%	−0.8	260	18 h
Ni/Fe–N–C	0.5 M KHCO_3_	CO	98%	−0.7	7.4	30 h
H–NiFe/NG	0.1 M KHCO_3_	CO	94%	−0.8	18.2	20 h
PcCu-Fe-ZIF-8	0.1 M KHCO_3_	CO	98%	−0.7	7	10 h
^57^FeAg NPs	0.1 M KHCO_3_	CO	36%	−1.1	0.35	2.5 h
Fe–Co-ZIF	0.5 M KHCO_3_	CO, H_2_	93%	−0.55	8	10 h
CuFe/OG	—	CH_4_	—	0.97	—	—
FeNi/DG	—	CH_4_	—	−0.44	—	—
FeCo-Pc	—	C_2+_	—	−0.66	—	—

## 13. Summary and outlook

Possibilities for the advancement of sustainable energy technology look promising for future studies on electrochemical CO_2_ reduction with bimetallic catalysts. Optimizing catalyst compositions and structures to increase selectivity and efficiency in the production of CO, syngas, and other multi-carbon products is a crucial field of research. Fe–Ni, Fe–Ag, Fe–Mo, Fe–Co, Cu–Fe, and Fe/Mn–N are examples of novel metal combinations that present the potential for enhanced catalytic performance. The main goals of the research will be to comprehend the fundamental structure–activity correlations and stability of these bimetallic catalysts in practical working environments. The scalability of bimetallic catalysts for large-scale commercial applications is limited by their typical synthesis, which involves intricate deposition, pyrolysis, and reduction methods. The development of more affordable, optimized synthesis techniques with improved loading capacities is necessary to meet this challenge and permit Fe-based bimetallic catalysts to be widely used in renewable energy systems. Moreover, a major challenge presented by the chemical instability of these catalysts is the reduction of active sites and changed performance caused by corrosion of the carbon substrate. Under practical circumstances, Fe-based bimetallic electrocatalysts for CO_2_ reduction encounter difficulties such as low catalytic activity, poor selectivity for target products, and restricted stability. Controlling the chemical intermediates, improving the electrical and geometric properties, and interpreting the synergistic effects between metals are still major challenges. Precise control of Fe-based bimetallic catalysts' shape, structure, and atomic coordination is required to strike a compromise between stability and catalytic activity. The aim is to design specialized bimetallic catalysts with enhanced stability features and active sites outperforming existing catalysts. This will facilitate the development of scalable CO_2_ conversion technologies for use in sustainable energy applications, assisting in the shift to a world without carbon emissions. The development of effective CO_2_ electroreduction catalysts will be speed up by collaborative, multidisciplinary research that combines theoretical and experimental methods. Future research should concentrate on investigating novel bimetallic combinations that improve performance and customizing catalyst structures by nano-structuring. Enhancing these catalysts' scalability for industrial applications is also essential. Developments in reaction mechanism research, computational modeling, and *in situ* characterization methods will improve catalyst design and propel more effective CO_2_ conversion systems.

## Data availability

No primary research results, software, or code have been included, and no new data were generated or analyzed as part of this review.

## Author contributions

Ayesha Zafar: writing – original draft. Adnan Majeed: writing – review & editing and software. Abdul Ahad: formal analysis. Muhammad Adnan Iqbal: conceptualization, resources, supervision. Tanveer Hussain Bokhari: validation. Zanira Mushtaq: data curation, validation. Shahzaib Ali: visualization.

## Conflicts of interest

The authors declare no conflict of interest.
